# The German adaptation of the Cambridge pulmonary hypertension outcome review (CAMPHOR)

**DOI:** 10.1186/1477-7525-10-110

**Published:** 2012-09-13

**Authors:** Katharina Cima, James Twiss, Rudolf Speich, Stephen P McKenna, Ekkehard Grünig, Christian M Kähler, Nicola Ehlken, Ursula Treder, Sigrid R Crawford, Lars C Huber, Silvia Ulrich

**Affiliations:** 1 Department für Innere Medizin, Schwerpunkt Pneumologie, Universitätsklinik, Innsbruck, Austria; 2 Galen Research Ltd, Manchester, UK; 3 Pulmonary Hypertension Program, University Hospital, Zurich, Switzerland; 4 Thoraxklinik, Universitätsklinikum, Heidelberg, Germany

**Keywords:** Pulmonary hypertension, Pulmonary arterial hypertension, Chronic thromboembolic pulmonary hypertension, Quality of life, Patient reported outcome, German adaptation

## Abstract

**Background:**

Individuals with precapillary pulmonary hypertension (PH) experience severely impaired quality of life. A disease-specific outcome measure for PH, the Cambridge Pulmonary Hypertension Outcome Review (CAMPHOR) was developed and validated in the UK and subsequently adapted for use in additional countries. The aim of this study was to translate and assess the reliability and validity of the CAMPHOR for German-speaking populations.

**Methods:**

Three main adaptation stages involved; translation (employing bilingual and lay panels), cognitive debriefing interviews with patients and validation (assessment of the adaptation’s psychometric properties). The psychometric evaluation included 107 patients with precapillary PH (60 females; age mean (standard deviation) 60 (15) years) from 3 centres in Austria, Germany and Switzerland.

**Results:**

No major problems were found with the translation process with most items easily rendered into acceptable German. Participants in the cognitive debriefing interviews found the questionnaires relevant, comprehensive and easy to complete. Psychometric analyses showed that the adaptation was successful. The three CAMPHOR scales (symptoms, activity limitations and quality of life) had excellent test-retest reliability correlations (Symptoms = 0.91; Activity limitations = 0.91; QoL = 0.90) and internal consistency (Symptoms = 0.94; Activity limitations = 0.93; QoL = 0.94). Predicted correlations with the Nottingham Health Profile provided evidence of the construct validity of the CAMPHOR scales. The CAMPHOR adaptation also showed known group validity in its ability to distinguish between participants based on perceived general health, perceived disease severity, oxygen use and NYHA classification.

**Conclusions:**

The CAMPHOR has been shown to be valid and reliable in the German population and is recommend for use in clinical practice.

## Introduction

Precapillary pulmonary hypertension (PH) consists of the WHO clinical classes 1 and 4, i.e. pulmonary arterial hypertension (PAH) and chronic thromboembolic PH (CTEPH). Despite many new therapeutic options (with the exception of patients with operable CTEPH) life expectancy is still dramatically shortened [1-3]. Hence, the search for new therapies is on-going, and multiple trials are underway or being planned. One of the main problems of planning such large trials is the absence of an ideal endpoint [4]. Invasive haemodynamic parameters are considered to represent hard endpoints, which are optimal for phase II trials. However, it is not feasible to perform these measurements repeatedly in everyday clinical practice. Most trials now rely on the 6-minute walking distance (6MWD) as the primary endpoint. However, this test has many flaws including disagreement about the normal values, the quality with which it is conducted, high standard deviation of distances walked and large minimally important difference of over 40 meters [5]. In reality, exercise testing does not provide an accurate reflection of how patients really feel.

As a consequence, quality of life (QoL) has emerged as a potentially important endpoint. Most clinical studies to date have used generic patient-reported outcome measures such as the SF-36 [6-9], the EuroQol [9,10] or the Nottingham Health Profile [11]. However, generic instruments generally have a low responsiveness, particularly with PH patients [12]. For instance, the minimally important difference of the SF-36 domains range between 13 and 25 points on a 100-point scale [12]. This implies that scores have to improve by up to 25 points before the change is noticeable by patients. Some trials have applied a modified version of the Minnesota Living with Heart Failure (MLWHF) questionnaire [13,14]. Although this has been shown to have a reasonable performance [15,16] it was not designed for patients with PH.

Given the impact of PH on morbidity, comprehensive disease-specific measures that directly address PH characteristics are needed. The Cambridge Pulmonary Hypertension Outcome Review (CAMPHOR) was designed as a disease-specific measure to assess both health-related QoL (symptoms and activity limitations) and QoL in patients diagnosed with PH [17]. It is an evaluation tool designed for use in clinical trials and clinical practice. The present paper describes the translation and validation of the CAMPHOR for use in German speaking countries. A successful adaptation will provide a valid and reliable outcome measure for use in PH clinical practice and clinical trials at German speaking centres.

## Methods

The adaptation of the CAMPHOR questionnaire was conducted in the three German-speaking countries; Austria, Germany and Switzerland. The process consisted of three main stages; translation (by means of bilingual and lay panels), cognitive debriefing interviews with patients to determine face and content validity and validation by means of a clinic visit and postal survey. Ethics committee approval was obtained from each participating centre and written informed consent was obtained from all participants.

### Step 1: Translation process

The bilingual translation panel involved a group of 2 Austrian, 3 German and 4 Swiss participants (7 females and 2 males with a median age of 47 and a range of 30 to 62 years) with good competence in both English and German who had the latter as their primary language. They were asked to consider the UK CAMPHOR instructions and items with the following requirements in mind; capturing the same concepts as the UK English version and producing a comprehensible and acceptable formulation of the concepts. Each item was discussed until agreement was reached. Where consensus could not be reached alternative versions of the item were taken forward for consideration by the lay panel.

A separate lay translation panel consisted of 1 Austrian, 2 German and 3 Swiss non-bilingual participants (3 females and 3 males; median age 27 ranging from 18 to 76 years). The purpose of this second panel was to ensure that the wording of items was appropriate for future respondents with average educational achievement. Participants were presented with the translations made by the bilingual panel and asked to comment on these in terms of comprehension and acceptability. In particular, they were asked to decide whether phrasing and language were acceptable or whether these should be changed to make the items more ‘natural’ while maintaining their original meaning. Where necessary they were also asked to choose between alternative translations that the bilingual panel had produced.

### Step 2: Cognitive debriefing interviews

Cognitive debriefing interviews were conducted with 4 PH patients from each country (Table 1). Eight of the interviewees had idiopathic pulmonary arterial hypertension (IPAH), 3 chronic thromboembolic pulmonary hypertension (CTEPH) and 1 had PH associated with scleroderma. The purpose of these interviews was to test the applicability, comprehension, relevance and comprehensiveness of the new scales with relevant patients. In the interviews, which were conducted face-to-face and semi-structured, the respondents were asked to complete the questionnaire in the presence of an interviewer and then to answer specific questions about the measure. Respondents were also asked whether any aspects of their experience of PH had been omitted.

**Table 1:** Demographic information

	**Cognitive debriefing interview panel (n = 12)**	**Validation sample (n = 107)**
**Gender**		
Male (%)	3 (25.0)	46 (43.4)
Female (%)	9 (75.0)	60 (56.6)
Missing	0	1
**Age (years)**		
Mean (SD)	60.2 (17.9)	60.3 (15.1)
Range	22 − 80	22–88
Missing	0	0
**Nationality**		
Swiss (%)	4 (33.3)	39 (36.4)
Austrian (%)	4 (33.3)	34 (31.8)
German (%)	4 (33.3)	34 (31.8)
Missing	0	0
**Marital status**		
Married/living as married (%)	7 (58.3)	68 (64.2)
Living alone (%)	5 (41.7)	38 (35.8)
Missing	0	1
**Employment status**		
Working (%)	3 (25.0)	21 (19.8)
Not working (%)	9 (75.0)	85 (80.2)
Missing	0	1

### Step 3: Validation

To validate the German version of the CAMPHOR, 107 PH patients from the three participating centres were recruited (Tables 1 and 2). During a visit to the outpatient clinic questions about demography (sex, age, marital status, occupation) and current condition (time since diagnosis, duration of symptoms, oxygen use and perceived general health) were asked. During the visit a 6MWT also was performed according to current guidelines [18]. This measures the distance a patient can walk quickly on a flat, hard surface in 6 minutes. It is intended to indicate functional capacity. Its reliability has been shown to be good in a number of different diseases [19-21]. The measure has also been shown to correlate with a number of PH related outcomes [22,23]. 

**Table 2:** Validation sample; disease information (n = 107)

**Cause of PH**	
Idiopathic PAH (%)	47 (45.2)
Associated PAH (%)	24 (21.2)
CTEPH (%)	36 (33.6)
Missing	0
**Duration of PH (years)**	
Mean (SD)	4.0 (4.5)
Range	0.2–25.0
Missing	6
**Hospital admission in last year due to PH**	
Yes (%)	4 (3.8)
No (%)	101 (96.2)
Missing	2
Oxygen	
Yes (%)	40 (37.7)
No (%)	66 (62.3)
Missing	1
**NYHA Classification**	
I (%)	3 (2.8)
II (%)	42 (39.3)
III (%)	56 (52.3)
IV (%)	6 (5.6)
Missing	0
**6 Min Walk Test (metres)**	
Mean (SD)	447.9 (112.7)
Range	180–660
Missing	8
**Perceived general health**	
Very good/Good (%)	65 (62.5)
Fair (%)	28 (26.9)
Very poor/ Poor (%)	11(10.6)
Missing	3
**Perceived severity of PH**	
No symptoms (%)	2 (1.9)
Mild (%)	23 (22.3)
Moderate (%)	55 (53.4)
Very & Quite severe (%)	23 (22.3)
Missing	12

NYHA functional class was determined at the outpatient visit (Class 1, no functional limitation; Class 2, slight functional limitation; Class 3, marked functional limitation; Class 4, inability to perform functions without symptoms) [24].

Patients also completed the Nottingham Health Profile (NHP; [25]).

The CAMPHOR was then administered on two occasions by mail approximately two weeks apart.

### Statistical analyses

Data are presented as means and standard deviations (SD) for illustrative purposes. Non-parametric statistical tests were used throughout the analyses due to the ordinal nature of the measures employed. All statistical tests were two-tailed with a *P* value of < .05 indicating statistical significance. Internal consistency of the CAMPHOR adaptations was evaluated by determining Cronbach’s alpha coefficients. The relatedness of individual items to the overall score was also assessed using the corrected item-total correlation coefficients. Test-retest reliability (patient-specific agreement between the two repeated administrations) was examined using Spearman’s rank correlations. Construct validity was assessed with the NHP as the comparator instrument.

Known-group validity (ability to distinguish between groups of patients who differed according to some known factor) was tested for:

Perceived general health (very good/good; fair; poor/very poor)

Perceived PH severity (No symptoms/mild; Moderate; Quite severe/very severe)

Oxygen use (yes / no)

NYHA class (NYHA I and II versus NYHA III and IV)

### Outcome measures

#### CAMPHOR

The CAMPHOR was originally developed and validated in the United Kingdom [17]. It has subsequently been adapted for use in the US [26], Canada (French and English) [27], Australia / New Zealand [28] and Sweden. It consists of a 25-item symptoms scale (scored 0–25), a 15-item functioning scale (scored 0–30) and a 25-item QoL scale (scored 0–25). For all scales, a low score indicates a better status. All language versions have been shown to have good internal consistency, reproducibility and validity [26-28].

#### Nottingham Health Profile

The NHP is a 38-item measure of perceived distress that has been widely used in health research [25]. It consists of six sections that assess; energy level, pain, emotional reactions, sleep, social isolation and physical mobility. All sections are scored 0 to 100 with a low score indicating good health status.

The NHPD is a 24-item unidimensional scale of impairment embedded in the NHP [29].

## Results

### Translation

No major difficulties were experienced during the translation process and most items in the questionnaire were translated without difficulty. Agreement was generally reached with little panel discussion. However, a small number of phrases required more extensive discussion. For example, “glücklich” was chosen instead of “zufrieden” for “happy” since the latter was considered to be closer to “satisfied” than “happy”. “Mein Zustand belastet mich” was preferred to “Mein Zustand nimmt mich mit” for “It gets me down” because the former was thought to represent the burden of the disease better. “Zur Zeit” was favoured over “zum jetzigen Zeitpunkt” for “at the moment”, as the latter was believed to be more accurate.

### Cognitive debriefing interviews

The CAMPHOR was completed in a median of 15 (range 7 to 25) minutes. Based on their responses participants found the questionnaire to be clear, unambiguous, comprehensive and easy to complete. Only minor difficulties were reported. For example, one patient had difficulty understanding one of the items and another thought that a different item was not relevant. None of the patients judged the questionnaire to be inappropriate or difficult to answer. Three patients thought that an additional item should be added to the questionnaire. Each suggested a different item and none were relevant to the content of the scales. No changes were required to the questionnaire as a result of the cognitive debriefing interviews. However, despite the instructions, only two respondents checked that they had answered all questions after completing the measure. Consequently, the instructions were highlighted in an attempt to overcome this oversight.

### Validation

One-hundred and seven participants were recruited at Time 1. At Time 2, 97 of these (90.7%) completed and returned the questionnaires.

### Sample demographics

Participant details are shown in Table 1. Individual country sample sizes were insufficient to permit assessment of the national psychometric properties separately and the samples were merged for the analysis. Disease information for the sample is shown in Table 2.

### Questionnaire descriptive scores

The missing response rate at the item level on both the CAMPHOR and NHP varied between 2 and 3%.

CAMPHOR scores at Time 1 are shown in Table 3. The scores for the CAMPHOR were relatively low suggesting that the sample had relatively mild PH. Floor effects (>10% of patients scoring minimum) were evident for the QoL scale. This reflects the mild nature of the sample.

**Table 3:** Questionnaire descriptive scores Time 1 (n = 107)

	**Mean (SD)**	**Median**	**IQR**	**Range**	**% scoring minimum**	**% scoring maximum**
**CAMPHOR**						
Symptoms	7.4 (6.5)	5.5	2–13	0–25	8.4	0.9
Activity limitations	8.7 (6.3)	8.0	3–13	0–29	6.5	0
QoL	5.1 (6.0)	3.0	1–7	0–24	21.5	0
**NHP**						
Energy level	38.6 (38.3)	33.3	0–66.7	0–100	37.4	18.7
Pain	10.6 (19.8)	0.0	0–12.5	0–100	60.7	0.9
Emotional reactions	13.0 (18.1)	0.0	0–22.2	0–78	48.6	0
Sleep	24.5 (29.1)	20.0	0–40	0–100	42.1	2.8
Social isolation	5.2 (13.7)	0.0	0	0–80	80.4	0
Physical mobility	22.3 (21.0)	12.5	0–37.5	0–100	29.0	0.9
NHPD	3.4 (3.6)	2.0	0–6	0–14	28.0	0

### Internal consistency and reproducibility

Internal consistency and test-retest reliability (reproducibility) are shown in Table 4. Reproducibility was above the required 0.85 level for all three scales. Internal consistency (Cronbach’s alpha) coefficients for the three scales were above the minimum required 0.7 indicating adequate inter-relatedness of items in each scale. The coefficients were above 0.9 for all CAMPHOR scales suggesting possible redundancy of items. The corrected item-total correlation coefficients (CITCC) were examined for all three scales to determine if there were any redundant items (items outside the 0.2 – 0.8 range). None of the items had CITCC’s above 0.8. In addition, the deletion of any one item would not have decreased the Cronbach’s alpha value significantly.

**Table 4:** Internal consistency and reproducibility of the CAMPHOR scales adaptations (n = 107)

	**Symptoms**	**Activity limitations**	**QoL**
Internal consistency	.94	.93	.94
Test-retest reliability	.91	.91	.90

### Convergent validity

Table 5 shows correlations between the CAMPHOR, NHP and the 6MWT at Time 1. The symptoms scale showed strongest correlations with the emotional reactions, energy level and physical mobility sections of the NHP. It is known that PH has a close relation to these outcomes. The activity limitations scale most closely related to physical mobility and energy level. Again this would be expected as the activity limitations scale assesses physical mobility and energy level is also closely related to mobility. For the same reason the 6MWT had the highest correlation with the activity limitations scale of the CAMPHOR. QoL was clearly influenced by all issues covered by the NHP but less so by pain and social isolation.

**Table 5:** Convergent validity CAMPHOR scales and NHP sections (n = 107)

	**Symptoms**	**Activity limitations**	**QoL**
NHP-Energy level	.82	.66	.71
NHP-Pain	.45	.56	.54
NHP-Emotional reactions	.74	.59	.72
NHP-Sleep	.50	.39	.51
NHP-Social isolation	.39	.32	.38
NHP-Physical mobility	.74	.76	.70
NHP-D	.81	.66	.72
6 Minute Walk Test (meters)	-.43	-.56	-.41

All correlations were significant at p < .01.

### Association of CAMPHOR scores with demographic factors

There were no significant differences in mean CAMPHOR scores related to gender with the exception of the Activity limitations scale where females scored higher than males (means = 9.3 and 7.8 respectively, p < .05). Older individuals (above median age) scored higher on the Symptoms scale than younger respondents (means = 5.9 and 9.1 respectively, p < .05) and Activity limitations scale (means = 6.7 and 10.9 respectively, p < 0.001). There were no differences in CAMPHOR scale scores related to duration of PH.

### Known group validity

Known groups validity results are shown in Table 6. All three CAMPHOR scales were able to discriminate between patients based on their perceived general health and severity of PH. Individuals with worse general health and worse PH had higher scores for the Symptoms, Activity limitations and QoL scales. All three CAMPHOR scales were able to distinguish between participants based on whether or not participants received oxygen for their PH.

**Table 6:** CAMPHOR scale scores by disease factors (n = 100-102)

	**Symptoms**			**Activity limitations**			**QoL**		
	**n**	**Mean**	**(SD)**	**n**	**Mean**	**(SD)**	**n**	**Mean**	**(SD)**
**Perceived general health**									
Very good/good	64	4.4	(4.3)	63	6.1	(5.2)	64	2.7	(3.8)
Fair	26	10.7	(5.8)	28	10.9	(5.3)	25	7.6	(6.8)
Poor/very poor	11	17.6	(3.5)	11	17.1	(4.9)	11	13.3	(5.3)
p		<.001			<.001			<.001	
**Perceived severity of PH**									
No symptoms / mild	25	3.7	(3.4)	25	5.3	(4.3)	25	2.3	(2.7)
Moderate	53	5.9	(5.4)	54	7.4	(5.5)	53	3.9	(5.4)
Quite / very severe	23	15.2	(4.6)	23	15.1	(5.3)	22	11.1	(6.1)
p		<.001			<.001			<.001	
**Oxygen use**									
Yes	38	9.8	(6.8)	37	11.6 (6.7)		37	7.3	(6.5)
No	63	5.9	(5.7)	65	7.0 (5.5)		63	3.6	(5.0)
p		.003			<.001			.001	

**Figure 1:**
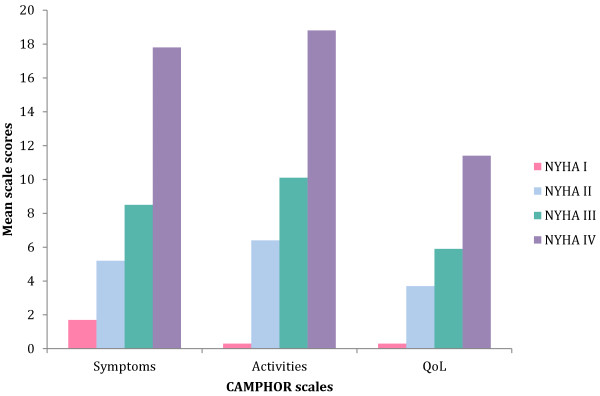
Mean CAMPHOR scales scores by NHYA classifications.

Figure 1 shows CAMPHOR scores by NYHA classification. The figure shows that the CAMPHOR was able to discriminate well between patients. Due to the small number of patients in classes I and IV statistical analyses compared respondents in NYHA groups I and II combined with participants in groups III and IV combined. Individuals in groups NHYA III & IV had higher CAMPHOR scores than those in NHYA I and II (Symptoms p < .001; Activity limitations p < .001; QoL p < .05).

## Discussion

The results showed that the adaptation of the CAMPHOR for use with German speaking participants was successful. Few problems were encountered during the translation process. All participants in the cognitive debriefing panel reported that the measure was easy to complete and that it covered important aspects of their experience of PH.

All scales in the CAMPHOR had good internal consistency and reproducibility. The CAMPHOR showed expected levels of association with the different NHP sections. Further evidence of the validity of the German CAMPHOR was shown by its ability to distinguish between groups known to differ by perceived disease severity, general health and oxygen use. Importantly, clinical validity was also demonstrated; CAMPHOR scores were moderately related to the 6MWT and were able to distinguish between NYHA classifications. On the basis of these findings clinicians working in the three German-speaking nations can have confidence in CAMPHOR scale scores.

One of the strengths of the study is that it tested the CAMPHOR in 3 German-speaking countries. In all these countries written German is the same modern Standard German known as High German (Hochdeutsch). Most non-German researchers may not consider the differences between the German speaking nationalities and assume that an adaptation in one country will be suitable for all German speakers. However, the language used in the CAMPHOR is, intentionally, as informal as possible and it is possible that this could have led to differences in wording in the different countries. Variations between the German dialects are considerable and often only the neighbouring dialects are mutually intelligible. Low German, most Upper German and even some Central German dialects when spoken in their purest form are not intelligible to people who only know standard German. The German dialects of South Tyrol have been influenced by local Romance languages with many words borrowed from Italian. Furthermore, there is no standard Swiss German. Valais German differs markedly from Bernese German. Hence, adapting the CAMPHOR in relatively central cities in the three countries should guarantee a broad understandability.

The sample included in this study appeared to be less severe than those commonly included in clinical trials and in the UK validation survey. However, this study was designed to test the performance of the German CAMPHOR rather than to report on the impact of PH on QoL. Future studies can be undertaken to relate PH variables to QoL.

Increasingly, health authorities are conducting economic evaluations in health care. High-cost orphan diseases like PH are prone to careful scrutinisation. The preferred methodology is cost utility analysis (CUA) whereby the benefits of health care interventions are measured according to quality adjusted life years (QALYs). Due to their lack of sensitivity and relevance, the commonly used generic preference based measures of health status may be inappropriate for some specific clinical conditions such as PH. The CAMPHOR Utility Index embedded in the CAMPHOR QoL scale permits derivation of PH-specific utility scores [30]. In contrast to traditional outcomes, such as NYHA functional class, 6MWD and generic QoL instruments, it has been shown to be valid and responsive to change [31]. Further studies testing the use of the CAMPHOR utility index with German speaking PH patients would be beneficial.

In conclusion, the current study shows that the German translation of the CAMPHOR is a valid and reliable instrument for assessment of health-related QoL and QoL in patients with precapillary PH. Its use in clinical practice can be recommended.

## Competing interests

None of the authors have any competing interests.

## Authors information

The CAMPHOR is a copyrighted instrument that is available free of licensing fees for use by clinicians and researchers in non-commercial studies. A small administrative fee is charged for such use. Readers interested in using the CAMPHOR should contact Stephen McKenna on smckenna@galen-research.com or http://www.galen-research.com

## Authors’ contributions

KC contributed to data acquisition, data analysis and drafting of the article. JT and SPM, contributed to study design, data collection, data analysis and interpretation of results. RS contributed to study design, data collection, data analysis, and drafting of the manuscript. EG, CMK, NE, UT contributed to data acquisition. SRC contributed to data analysis and interpretation of results. LCH contributed to data analysis and drafting of the manuscript. SU contributed to study design and data acquisition. All authors were involved with the critical revision of the manuscript.

## Author details

^1^Department für Innere Medizin, Schwerpunkt Pneumologie, Universitätsklinik, Innsbruck, Austria. ^2^Galen Research Ltd, Manchester, UK.^3^Pulmonary Hypertension Program, University Hospital, Zurich, Switzerland.^4^Thoraxklinik, Universitätsklinikum, Heidelberg, Germany.
